# The Power of Representation: Advancing Health Equity for African American Men Across the Life Course

**DOI:** 10.1093/geroni/igaf122.1530

**Published:** 2025-12-31

**Authors:** Roland Thorpe

**Affiliations:** Johns Hopkins Bloomberg School of Public Health, Baltimore, Maryland, United States

## Abstract

Roland Thorpe, Ph.D. is a Professor in the Department of Health, Behavior, and Society in the Bloomberg School of Public Health where he also serves as Deputy Director of the Hopkins Center for Health Disparities Solutions, and a Director of the Johns Hopkins Alzheimer’s Disease Resource Center for Minority Aging Research. He holds joint appointments in the Division of Geriatric Medicine and Gerontology and the Department of Neurology in the School of Medicine, and the Department of Sociology in the Krieger School of Arts and Sciences. Dr. Thorpe is a social epidemiologist and gerontologist, whose research focuses on how race, socioeconomic status, and segregation influence health and well-being for African Americans, particularly African American men. His work has been funded by the NIH and he has published extensively. He is the recipient of numerous awards that recognize his commitment and valuable contributions to student and faculty mentoring, including the Johns Hopkins Bloomberg School of Public Health Advising, Mentoring, and Teaching Recognition Award, the inaugural 2018 NHLBI OHD PRIDE Roland J. Thorpe, Jr. Mentoring Award, the 2020 Johns Hopkins Bloomberg School of Public Health Dean’s Award of Distinction in Faculty Mentoring, and the 2020 Minority Issues in Gerontology Outstanding Mentorship Award. Dr. Thorpe earned a bachelor’s in theoretical mathematics from Florida A&M University, a master’s in statistics, and a Ph.D. in clinical epidemiology with a graduate minor in gerontology from Purdue University.

